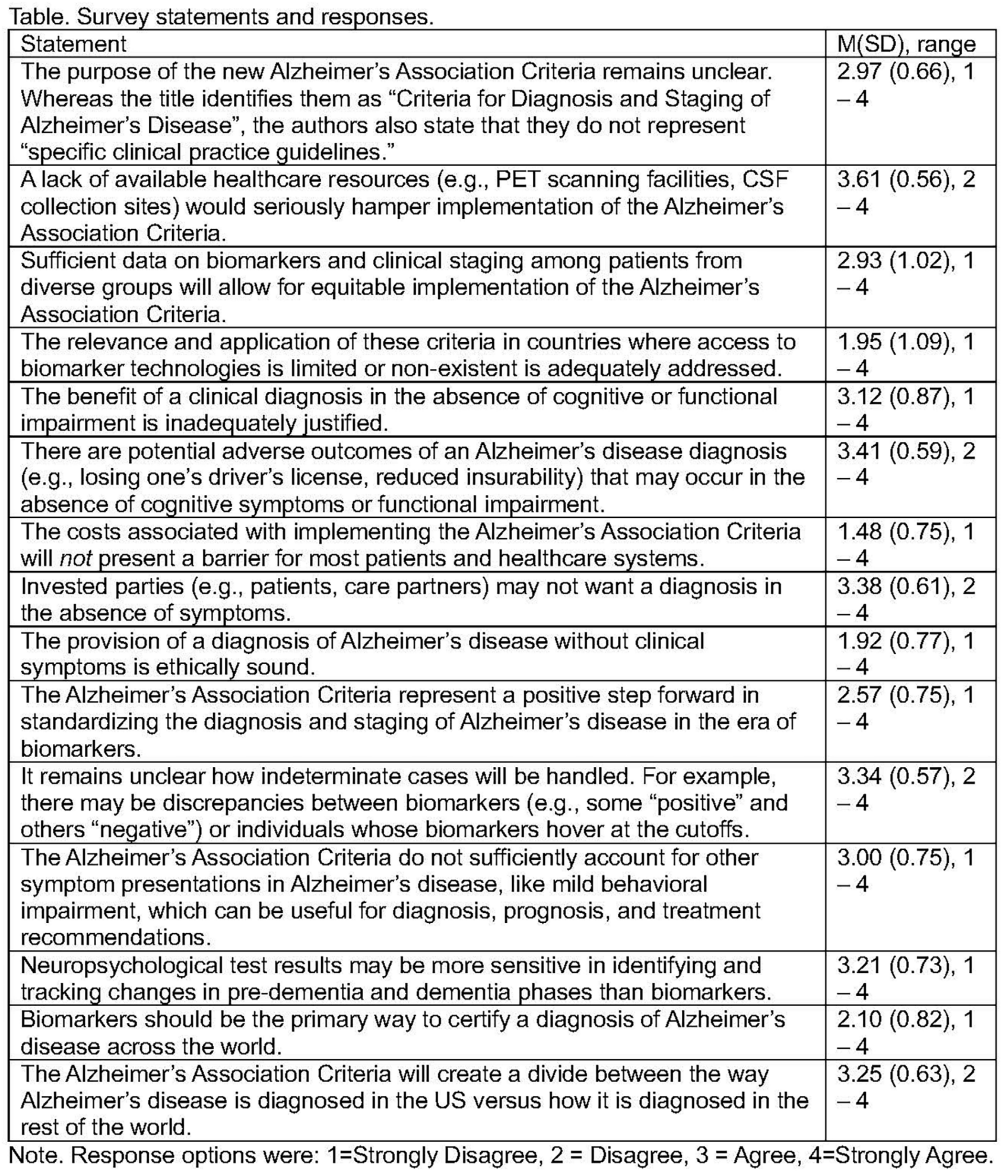# Reactions to the proposed diagnostic criteria on Alzheimer’s disease from clinical neuropsychologists who specialize in dementia

**DOI:** 10.1002/alz.095578

**Published:** 2025-01-09

**Authors:** Kevin Duff, Andrew M Kiselica, Marissa A. Gogniat, Unai Diaz‐Orueta, S Duke Han

**Affiliations:** ^1^ Oregon Health & Science University, Portland, OR USA; ^2^ NIA‐Layton Aging & Alzheimer’s Disease Research Center, Portland, OR USA; ^3^ University of Missouri, Columbia, TX USA; ^4^ University of Pittsburgh School of Medicine, Pittsburgh, PA USA; ^5^ Maynooth University, Maynooth Ireland; ^6^ University of Southern California, Leonard Davis School of Gerontology, Los Angeles, CA USA

## Abstract

**Background:**

The Alzheimer’s Association (AA) is updating existing recommendations for the diagnosis of Alzheimer’s disease (AD) to focus on biomarkers, independent of clinical syndrome. Although this perspective is consistent some other medical conditions (e.g., cancer) and has merit in research settings, there is concern about how the proposed approach will be implemented in clinical practice and how it might affect patients and families. Its on existing inequities in the diagnosis and treatment of AD are unclear.

**Methods:**

Members of the Dementia Special Interest Group of the International Neuropsychological Society were surveyed about their opinions on these AA recommendations. The survey queried members about their demographics and asked them to rate their agreement with 15 statements related to the proposed recommendations (e.g., purpose of the recommendations, applicability to diverse groups in and outside of the United States, access to biomarker testing). The 15 statements were rated on a 4‐point Likert scale (1 = Strongly Disagree to 4 = Strongly Agree), with six reverse‐scored.

**Results:**

Sixty‐one respondents (67% female, 84% non‐Hispanic, 79% white, 75% from United States) provided analyzable data. For 67% of statements, the full range of responses (i.e., 1 – 4) were observed. After recoding the statements, higher values reflected greater concern regarding the recommendations. The mean rating was 3.12 (SD = 0.36), suggesting that, overall, respondents had concerns about the recommendations. Statements that yielded the most concern focused on barriers to implementing the recommendations, including lack of healthcare resources and costs of biomarker testing (mean ratings: 3.52 – 3.61). Conversely, participants viewed updated diagnostic criteria as a positive step forward for the field and were equivocal about the applicability of criteria to diverse groups (mean ratings: 2.07 – 2.43).

**Conclusions:**

In this survey of neuropsychologists and trainees who focus on dementia, there was concern about the proposed recommendations for diagnosing AD mainly on biomarkers. Limitations of this study included small sample size, ambiguous wording of some questions, and lack of a ”neutral” response option. In conclusion, although reactions were varied, there is clear concern about how the AA’s proposed approach to diagnosing AD would be implemented both inside and outside of the United States.